# Distribution of insertion sequences associated with Tn1546 and clonal diversity of vancomycin-resistant enterococci isolated from patients in Tehran, Iran

**Published:** 2010-03

**Authors:** M Oskoui, P Farrokh

**Affiliations:** 1 Department of Bacteriology, Pasteur Institute of Iran, Tehran, Iran

**Keywords:** Vancomycin- resistant enterococci, Tn *1546*, insertion sequences, Iran

## Abstract

**Background and objectives:**

Infection with vancomycin-resistant enterococci (VRE) has caused a therapeutic problem. VanA and VanB resistant types are the predominant phenotypes among vancomycin resistant enetrococci. Transposon 1546 (Tn*1546*) harboring the *van*A gene cluster, plays an important role in the horizontal transfer of *van*A gene. In this study, we examined the phenotypic and genotypic diversity of a number of clinical VRE.

**Materials and Methods:**

Twenty-four clinical VRE isolated from two university hospitals in Tehran were examined based on their antimicrobial susceptibility, Tn*1546* related element organization and pulsed-field gel electrophoresis (PFGE) patterns. Integration of well-studied insertion sequence elements IS*1216V*, IS*1542* and IS*1251* was examined by PCR mapping and sequencing.

**Results:**

From 24 isolates, 15 isolates with VanA phenotype and 9 isolates with VanB phenotype were identified which both groups interestingly possessed the *vanA* gene. According to PCR mapping, our isolates were assigned to 6 main groups. In 14 (58.3%) isolates, IS*1216V* was inserted in *vanX*-*vanY* region and/or in truncated left-hand of Tn*1546*-like elements. In 11 (45.8%) isolates, both IS*1216V* and IS*1542* were inserted in *vanX*-*vanY* and *orf*2-*vanR* regions, respectively and none of them harbored IS*1251*. Interestingly, PFGE of the isolates showed a high degree of diversity.

**Conclusion:**

PCR mapping revealed that VanA elements in our isolates were highly heterogeneous. Overall, we found no correlation between transposon type and PFGE pattern. Genetic diversity of VRE provides practical information for epidemiological studies and our data showed horizontal transfer of VRE in this region.

## INTRODUCTION

Enterococci are part of the normal flora in the gastrointestinal tracts of humans (1, 2); however, they can also be the main cause of nosocomial infection especially in immunocompromised patients (3, 4). Vancomycin-resistant enterococci (VRE) have presented a global problem for treatment (3, 5, and 6). VRE are phenotypically and genotypically heterogenous (7) and there are six types of glycopeptide resistance in enterococci (VanA to VanE and VanG) (8, 9). VanA phenotype with acquired inducible resistance to both vancomycin and teicoplanin, and VanB phenotype with variable level of resistance to vancomycin but susceptibility to teicoplanin are the most predominant ones (10, 11). Recently, VRE with *vanA* genotype, susceptible to teicoplanin (VanB phenotype-*vanA* genotype), has become increasingly prevalent in Asia (11).

VanA gene cluster is carried on transposon Tn*1546* or closely related elements (5, 12) consisting of *vanR*, *vanS*, *vanH*, *vanA*, *vanX*, *vanY*, *vanZ* genes (3). *orf*1 and *orf*2 with transposition function are also present in the left end of Tn*1546*. Due to integration of insertion sequences (ISs), point mutation, and deletions in nonessential genes or integration regions, there are considerable variations among Tn*1546* elements (4, 5, 12).

Investigation of IS elements in VanA gene cluster provides useful information for epidemiological studies of the dissemination of VRE due to horizontal transfer of Tn*1546*-like elements (3, 13). The most common insertion sequences reported in *vanA* gene cluster are IS*1216V*, IS*1542*, IS*1251*, and IS*1476* (14). Although IS*1216V* is known in most of the *vanA* elements, the other IS elements appear to be geographically restricted (14). Beside horizontal transfer of resistance gene cluster, clonal dissemination of VRE was determined in various studies (14, 15).

Although vancomycin-resistant enterococci have been reported worldwide, investigation of resistant isolates from different geographic locations provides useful information (16, 17). In this study, our goal is determination of the phenotypic as well as genotypic diversity of clinical VRE in this region with using antimicrobial susceptibility, PCR mapping and Pulse-field gel electerophoresis.

## MATERIALS AND METHODS

**Bacterial isolates and identification.** A total of 24 VRE clinical isolates, collected between May 2006 and May 2007 from Milad and Sina University Hospitals in Tehran, were studied. These VRE were isolated from nearly 500 enterococci. With the exception of isolates from blood samples, all enterococci were isolated from urine specimens. These isolates were identified by conventional biochemical reactions (18). *E. faecium* BM4147, *E. faecalis* ATCC 29212, and *E. faecalis* V583 were used as control strains.

**Antimicrobial agents and MIC determination.** Antimicrobial susceptibility of the isolates were tested by the disc diffusion method and interpreted according to the Kerby-Bauer method (19). The antibiotics (MAST Diagnostics Ltd. Merseyside, England) used for disc diffusion assays included vancomycin (30µg), teicoplanin (30µg), tetracycline (30µg),erythromycin (15µg),chloramphenicol (30µg), ampicillin (10µg), and ciprofloxacin (5µg). Minimum inhibitory concentration (MIC) of vancomycin (SERVA FEINBIOCHEMICA GmbH & Co., Germany) and teicoplanin were determined using broth microdilution (19) and Etest (AB Biodisk, Solna, Sweden), respectively.

**Characterization of Tn** ***1546*****-like elements.**Extraction of chromosomal and plasmid DNA were performed using Bacterial Genomic and Plasmid Miniprep kits (Metabion, Martinsried, Germany). VanA and VanB-type enterococci were examined by PCR with primers specific for the *vanA* and *vanB* genes.

For structural analysis of Tn*1546*-like elements, PCR was carried out with previously published primers for *orf*1, *orf* 2, *orf* 2-*van*R, *van*S-*van*H, *van*A, *van*X-*van*Y, and *van*Z (Table 1). Presence of well- studied IS elements IS*1216V*, IS*1542* and IS*1251* in the *orf* 2-*vanR*, *vanS*-*vanH* and *vanX*-*vanY* intergenic regions were confirmed with additional PCR by one primer in the published sequence of Tn*1546* and one in the published IS sequence (Table 1). In order to determine precise left ends of the Tn*1546*, DNA fragments were amplified with a combination of Tn*1546*-derived primers and primers based on the IS*1216V* which was inserted at the left end of the transposon. The primer sequences and amplicon size of products are listed in Table 1.

**Table 1:** Nucleotide sequences of PCR primers used in this study.

Primer	Sequences(5’ to 3’)	Amplicon size (bp)	Reference
*orf* 1AF	ACGTTAAGAAAGTTTTAGTGG	1119	(27)
*orf* 1 AR	GCCCTTTTAGGAATGG		
*orf* 1 BF	CATACATGCGCCATTGAGATA	1696	(27)
*orf* 1 BR	GTTAGTCCATCCTCGCTTGAT		
IS1216V R	AGGATTATATAAGAAAACCCG	Variable	(14)
*orf* 1 BR	GTTAGTCCATCCTCGCTTGAT		(26)
*orf* 2 F	TTGCGGAAAATCGGTTATATTC	540	(26)
*orf* 2 R	AGCCCTAGATACATTAGTAATT		
IS1216V R	AGGATTATATAAGAAAACCCG	Variable	(14)
*orf* 2 R	GCCCTAGATACATTAGTAATT		(26)
IS1542 F	GAATCGCTTTTACTGCTTCTC	Variable	(25)
*van*R R	CGGATTATCAATGGTGTCGTT		(26)
*orf*_2_ F	TTGCGGAAAATCGGTTATATTC	Variable	(26)
IS1542 R	TTCTAAAGCTGCCATATTGC		
*orf*_2_ F	TTGCGGAAAATCGGTTATATTC	1485	(26)
*van*R R	CGGATTATCAATGGT GTC GTT		
*van*S F	TTGGTTATAAAATTGAAAAATAA	2338	(21)
*van*H R	TCCTTTCAAAATCCAAACAGTTT		
*van*S F	TTGGTTATAAAATTGAAAAATAA	Variable	(21)
IS1251 R	AGGATTATATAAGAAAACCCG		(3)
IS1251 F	TCAGACGACCTTGAGAAC	Variable	(3)
*vanH* R	TCCTTTCAAAATCCAAACAGTTT		
*vanA* F	ATGAATAGAATAAAAGTTGCAATAC	1029	(26)
*vanA* R	CCCCTTTAACGCTAATACGAT		
*vanX* F	ATGGAAATAGGATTTACTTT	1947	(21)
*vanY* R	TTACCTCCTTGAATTAGTAT		
*vanX* F	ATGGAAATAGGATTTACTTT	Variable	(21)
IS1216V F	AGGATTATATAAGAAAACCCG		(14)
IS1216*V* R	ACCTTCACGATAGCTAAGGTT	Variable	(14)
*vanY* R	TTACCTCCTTGAATTAGTAT		(21)
*vanZ* F	TTATCTAGAGGATTGCTAGC	454	(21)
*vanZ* R	AATGGGTACGGTAAACGAGC		
*vanB* F	CAAAGCTCCGCAGCTTGCATG	433	(7)
*vanB* R	TGCATCCAAGCACCCGATATAC		

PCR amplification was carried out on a Eppendorf thermal cycler with the following protocol: initial denaturation at 95°C for 4 min; this was followed by 30 cycle of DNA denaturation at 95°C for 30 S, primer annealing at 55-56°C for 1 min and DNA extension at 72°C for 1 min; and final extension at 72°C for 10 min.

**DNA sequence analysis.** PCR amplicons of *vanX*-*vanY* and *orf*2-*vanR* regions which were larger than those of the prototype *van*A gene cluster were sequenced with IS*1216V* and IS*1542* primers. To determine the DNA sequence of the left end of the VanA transposon derivatives, PCR products of this region were sequenced with IS*1216V* primers (Macrogen Research, Seoul, Korea).

**Pulse-field gel electerophoresis (PFGE).** PFGE was performed as described previously (20). Genomic DNA was digested with *SmaI* (Fermentas, Vilnius, Lithuania), and separated on a 1% agarose gel using a contour-clamped homogeneous-field apparatus (CHEF DR III system; Bio-Rad Laboratories, Richmond, CA). *Salmonella Braenderup* H9812 was used as molecular weight marker after *XbaI* (Fermentas, Vilnius, Lithuania) digestion. The agarose gels were run at 14°C and 6 V/cm for 21 h, with a linear pulse time of 5 to 40 sec at an angle of 120 degrees. The banding patterns were analyzed using Gelcompar II version 4.0 (Applied Maths Sint- Matens-Latem, Belgium).

## RESULTS

**Bacterial isolates and antibiotic resistance.** Eighteen Out of 24 VRE were *E. faecium* and the remaining were *E. faecalis*. All the isolates were resistant to vancomycin, ciprofloxacin and erythromycin discs. Furthermore, 79.2%, 66.7%,62.5% and 41.7% of the isolates were resistant to tetracycline,ampicillin,teicoplanin and chloramphenicol, respectively (Table 2). The MIC of vancomycin and teicoplanin of the isolates are given in Table 2. Fifteen out of 18 *E. faecium* showed the VanA phenotype, while all the *E. faecalis* and 3 of the *E. faecium* displayed VanB phenotype.

**Table 2:** The phenotypic and genetic characteristics of vancomycin resistant isolates of enterococci isolated from patients in Tehran.

Isolate	Source	MIC (µg/ml)		Van†		Resistance profile‡	Transposon Type

		Vancomycin	Teicoplanin	Phenotype	Genotype		
*E. faecium*							
B-201 IPI	urine	>256	>256	A	A	Vm, Tc, Cp, Em, Te	A_1_
495 IPI	urine	>256	128	A	A	Vm, Tc, Cp, Em, Te, Am, Cm	B_3_
105 IPI	urine	>256.	128	A	A	Vm, Tc, Cp, Em, Te, Am, Cm	A_1_
107 IPI	urine	>256	96	A	A	Vm, Tc, Cp, Em, Am, Cm	B_1_
431 IPI	urine	>256	64	A	A	Vm, Tc, Cp, Em, Te, Am, Cm	F_7_
B-364 IPI	urine	>256	64	A	A	Vm, Tc, Cp, Em, Te	F_6_
N-117 IPI	urine	>256	64	A	A	Vm, Tc, Cp, Em, Te, Am	C
557 IPI	urine	>256	64	A	A	Vm, Tc, Cp, Em, Te, Am, Cm	F_1_
390 IPI	urine	>256	64	A	A	Vm, Tc, Cp, Em, Te, Am, Cm	B_2_
110 IPI	urine	>256	64	A	A	Vm, Tc, Cp, Em, Am	B_1_
436 IPI	urine	>256	64	A	A	Vm, Tc, Cp, Em, Te, Am, Cm	E_1_
108 IPI	urine	>256	64	A	A	Vm, Tc, Cp, Em, Am, Cm	B_1_
B-269 IPI	urine	>256	64	A	A	Vm, Tc, Cp, Em	E_2_
102 IPI	urine	>256	48	A	A	Vm, Tc, Cp, Em, Te, Am	A_3_
B-148 IPI	blood	>256	32	A	A	Vm, Tc, Cp, Em, Te, Am	A_2_
106 IPI	urine	>256	1.5	B	A	Vm, Cp, Em, Te, Am, Cm	B_1_
109 IPI	urine	>256	1	B	A	Vm, Cp, Em, Te, Am	D
E-83 IPI	urine	>256	0.5	B	A	Vm, Cp, Em, Te	F_5_
*E. faecalis*							
523 IPI	urine	>256	1	B	A	Vm, Cp, Em, Te, Am	F_3_
N-89 IPI	urine	>256	0.5	B	A	Vm, Cp, Em, Te, Cm	F_2_
E-125 IPI	urine	128	0.5	B	A	Vm, Cp, Em, Te	F_8_
578 IPI	urine	32	0.38	B	A	Vm, Cp, Em	F_4_
524 IPI	urine	16	1.5	B	A	Vm, Cp, Em, Te, Am	F_10_
B-219 IPI	urine	16	1.5	B	A	Vm, Cp, Em, Te	F_9_

‡ Vm, Vancomycin; Tc, teicoplanin; Am, ampicillin; Cm, chloramphenicol; Cp, ciprofloxacin; Em, erythromycin; Te, tetracycline

**Structural analysis of Tn** ***1546*** **element by PCR mapping.** When PCR was carried out with the *van*A specific primer, PCR product of expected size was obtained from 15 isolates with VanA phenotype. None of the 9 isolates with VanB phenotype possessed the *vanB* gene, but all of them harbored the *vanA* gene.

PCR mapping of Tn*1546*-like elements of 24 isolates revealed 6 main different transposon types (A-F) according to the patterns of ISs inserted into Tn*1546* (Table 3). Type A was characterized by an IS*1216V* insertion in the *vanX*-*vanY* intergenic region and an IS*1542* insertion in the *orf*2-*vanR* region. Type B was specified by the presence of two copies of IS*1216V* at the left-hand of Tn*1546* and the *vanX*- *vanY* region and an IS*1542* insertion in the *orf*2- *vanR* intergenic region. Type C was indicated with one copy of IS*1216V* in the left-hand of Tn*1546* and the presence of IS*1542* in the *orf*2-*vanR* intergenic region. Type D and E were characterized with one copy of IS*1216V* in the *vanX*-*vanY* region and one copy in the left-hand of Tn*1546*, respectively. Group F had no insertion sequences (Table 3).

**Table 3:** +, amplicons the same size to prototype, ++, amplicons larger than prototype and -, absence of an amplicon with particular primers.* PCR reaction was not performed.

PCR amplification of Tn1546-like elements															
		
**No. of isolates**	*orf*1A	*orf*1B	I*S*1216*V*R *orf*1BR	*orf*2	*S*1216*V*R *orf*2R	IS1542F *vanR*R	*orf*2F IS1542R	*orf*2F *vanR*R	*vanS*F*vanH*R	*vanA*	*vanX*F*vanY*R	IS1216*V*R*vanY*R	*vanX*F IS1216*V*F	*vanZ*	**Transposon Type**
**2**	+	+	—*	+	—	+	+	++	+	+	++	+	+	+	A_1_
**1**	+	+	—	+	—	-	+	-	+	+	++	+	+	+	A_2_
**1**	–	–	—	+	–	+	+	–	+	+	++	+	+	+	A_3_
**4**	–	+	+	+	—	+	+	++	+	+	++	+	+	+	B_1_
**1**	–	+	+	+	—	+	+	–	+	+	++	+	–	+	B_2_
**1**	–	+	+	+	—	–	+	–	–	+	–	–	+	+	B_3_
**1**	–	+	+	+	—	–	+	–	+	+	–	–	–	+	C
**1**	–	–	—	+	–	–	–	–	+	+	–	+	+	+	D
**1**	–	+	+	+	—	–	–	+	+	+	+	–	–	+	E_1_
**1**	–	–	—	+	—	–	–	+	–	+	–	–	–	+	E_2_
**1**	+	+	—	+	—	–	–	–	+	+	–	–	–	+	F_1_
**1**	+	+	—	+	—	–	–	+	–	+	–	–	–	+	F_2_
**1**	+	+	—	+	—	–	–	–	–	+	–	–	–	–	F_3_
**1**	–	–	—	+	–	–	–	+	–	+	+	–	–	–	F_4_
**1**	–	–	—	+	–	–	–	+	–	+	–	–	–	–	F_5_
**1**	–	–	—	+	–	–	–	–	–	+	+	–	–	+	F_6_
**1**	–	–	—	+	–	–	–	–	+	+	–	–	–	–	F_7_
**1**	–	–	—	+	–	–	–	–	–	+	–	–	–	+	F_8_
**1**	–	–	—	+	–	–	–	–	–	+	–	–	–	–	F_9_
**1**	–	–	—	–	—	–	–	–	–	+	–	–	–	+	F_10_

Through amplification of intergenic regions, only 9 and 6 isolates showed larger size of amplicon, approximately 3000bp, in the *vanX*-*vanY* and *orf* 2-*vanR* regions, respectively (Table 3).

**Sequence analysis of the VanA transposons.** Sequencing of *vanX*-*vanY* and *orf*2-*vanR* amplicons larger than those of the prototype VanA gene cluster (larger than 3000bp) showed that IS*1216V* and IS*1542* were inserted in these regions, respectively. We published partial sequence of IS*1216V* and IS*1542* with access numbers of FJ416860, FJ416861, GQ273971 and GQ273972 in GenBank (www.ncbi.nlm.nlh.gov/Genbank/submit.html). Sequencing of amplicons of the left end of Tn*1546*-like elements confirmed insertion of IS*1216V* in this region.

**Fig. 1:**
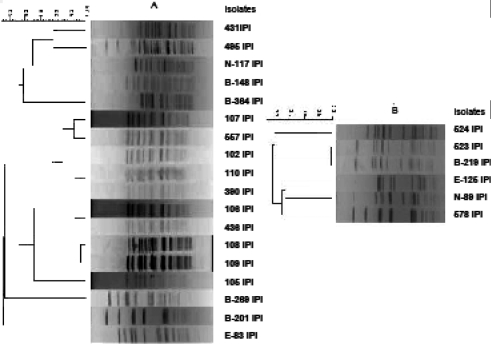
PFGE analysis of vancomycin resistant *E. faecium* (A) and *E. faecalis* (B). The phylogenetic tree is based on cluster analysis of the unweighted pair group method with average linkages (UPGAMA) and Dice analysis.

**PFGE profiles.** Analysis of the 18 *E. faecium* and 6 *E. faecalis* banding patterns differentiated 9 and 5 PFGE types respectively, with a similarity value of 0.7 (Fig. 1). So, the VRE isolates showed a high degree of heterogeneity. Among vancomycin resistant *E. faecium*, 9 isolates, with more than 78% similarity in their banding patterns, were the prevalent type.

## DISCUSSION

Up to now, phenotypic and genotypic varieties of the vancomycin-resistant enterococci have been investigated in several previous studies (13, 21). In the present study, we compared the phenotypic (antibiotic resistant patterns) as well as genotypic traits of 24 VRE, isolated from patients admitted in two major hospitals of Tehran. The predominant species in this study were *E. faecium* (75%) with *E. faecalis* accounting for 25% of the remaining isolates. Overall, the VRE isolates in spite of resistant to vancomycin, were 100% resistant to erythromycin and ciprofloxacin; furthermore, ampicillin showed a high frequency of resistance among *E. faecium* (94.5%). Similar to the previous study (22), chloramphenicol was the only drug that showed a lower rate of resistance among our isolates. Since consumption of antibiotics and the related selective pressures causes antibiotic resistance in community, it seems that high level of resistance to these antibiotics is related to their high consumption (1).

All the VRE harbored the *vanA* gene; however, 9 (37.5%) of them exhibited the VanB phenotype-*vanA* genotype. Incongruence of the VanB phenotype-*vanA* genotype has been found in Japan, Taiwan, and Korea (23), but to our knowledge, this is the first report of VanB-*vanA* VRE from humans in Iran.

According to PCR mapping, VanA elements were highly heterogeneous and none of them were completely similar to the prototype BM4147. Similar results were also obtained with previous studies (13, 14).

Based on PCR and sequencing results, IS*1216V* was found in 14 isolates (58.3%) in *vanX*-*vanY* and/or in the left-hand of Tn*1546*-like elements and had higher frequency among VRE. In 11 isolates (45.8%), both IS*1216V* and IS*1542* were inserted in *vanX*-*vanY* and *orf* 2-*vanR* regions. IS*1251* was identified neither with *vanS* F-*vanH* R primers nor with published primers of IS*1251* and *vanS*/*vanH* through PCR reaction. Thus, the distribution of ISs in Tn*1546*-like elements of our isolates is similar to those among Europeans and Koreans (2, 21, 24) but not to American VRE isolates (1, 12, 13).

*vanZ*, as a nonessential gene in Tn*1546,* was detected in 19 (79.2%) of our isolates using PCR. The *vanR*, *vanS*, *vanH* and *vanX* genes are essential for the expression of vanA resistance, therefore, it was surprising that some isolates lacked these amplicons after PCR with intergenic region primers. PCR results obtained from each single gene showed that in 100%, 87.5%, 79.2% and 62.5% of the isolates *vanR*, *vanS*, *vanH* and *vanX* were amplified, respectively (data was not shown). In this study, negative PCR reactions were repeated several times, but absence of PCR products may indicate disruptions or insertions in these regions and these kinds of difficulties with amplifying these regions of some VanA elements have been reported previously (25).

Many of the studied VanA elements (18 isolates) lacked one or more amplicons in the left-hand of Tn*1546*, corresponding to genes *orf* 1 and *orf* 2 associated with transposition functions. Similar findings were reported in Europe and Korea (14, 25).

Based on some reports, point mutation, insertion of IS elements and deletion of intergenic region can be responsible for the VanB phenotype-*vanA* genotype (2, 11) (23). It seems that loss of some Tn*1546* parts can be responsible for this change in our isolates.

Using PFGE we found high degree of diversity among the isolates. While 6 *E. faecium* with more than 78% similarity had both IS*1216V* and IS*1542* in *vanX*-*vanY* and *orf* 2-*vanR* regions, we generally found no correlation between the positions of Insertion sequences in the Tn*1546*-like elements and the isolates PFGE types. The genetic diversity among Tn*1546* elements was shown in several previous studies (14,24, and 25). The variability in transposon type in various countries may be explained by the different antibiotic selective pressures against glycopeptides (2, 25), as well as the geographic differences in the transposon distribution (14) and movement of ISs in Tn*1546* during outbreak (25).

In conclusion, our results revealed high degree of diversity and unique characterization among VRE in clinical specimens in this region. Thus, the genetic diversity indicated horizontal transfer of VRE rather than their clonal dissemination in this region and offered useful information for further epidemiological studies.
